# Low serum albumin and the risk of hospitalization in COVID-19 infection: A retrospective case-control study

**DOI:** 10.1371/journal.pone.0250906

**Published:** 2021-04-30

**Authors:** Roshan Acharya, Dilli Poudel, Aakash Patel, Evan Schultz, Michael Bourgeois, Rishi Paswan, Scott Stockholm, Macylen Batten, Smita Kafle, Amanda Atkinson, Hafiz Sarwar

**Affiliations:** 1 Department of Internal Medicine, Cape Fear Valley Medical Center, Fayetteville, NC, United States of America; 2 Department of Rheumatology, Indiana Regional Medical Center, Indiana, PA, United States of America; 3 RN-BSN Program, Fayetteville State University, Fayetteville, NC, United States of America; 4 Department of Emergency Medicine, Cape Fear Valley Medical Center, Fayetteville, NC, United States of America; 5 Department of Critical Care Medicine, Cape Fear Valley Medical Center, Fayetteville, NC, United States of America; Heidelberg University Hospital, GERMANY

## Abstract

**Background:**

The data on the COVID-19 patients who were discharged to self-quarantine is lacking.

**Aim:**

The aim of the study was to investigate the percentage of COVID-19 positive patients that were hospitalized within a three-week period after discharge from ED to self-quarantine.

**Methods:**

The patients who had confirmed SARS-CoV-2 on RT-PCR of the nasopharyngeal swab and were discharged from ED of a tertiary care hospital in the USA to self-quarantine from March 01- July 31, 2020, were included. Patients were divided into two groups based on serum albumin levels and were followed up for three weeks to see if low level of albumin increased the risk of hospitalization. Univariate and multivariate logistic regression analyses were performed to study the effect of albumin level and outcomes.

**Results:**

A total of 112 patients were included in the study out of which 65 had low serum albumin (<3.5 g/dL) and 47 had normal serum albumin (≥3.5 g/dL). More than 10% of patients discharged to self-quarantine needed hospitalization within three weeks. The Low albumin group had more co-morbidities at baseline. The low serum albumin group had 10 (15.38%) vs 2 (4.26%), p = 0.06 hospitalizations as compared to the normal serum albumin group. The multivariate logistic regression analysis did not reveal lower odds of hospitalization in the group with normal albumin, (OR 0.26, 95% CI 0.03–1.92, p = 0.19) after controlling for age, sex, and various co-morbidities.

**Conclusion:**

The low serum albumin was not associated with the risk of hospitalization in COVID-19 patients who were initially discharged to self-quarantine.

## Background

Coronavirus disease– 19 (COVID-19) was first reported almost a year ago [1]. Many studies have been done and much has been learned about the disease. The patients who have worse outcomes generally have overwhelming inflammatory response as evidenced by the presence of high levels of inflammatory markers such as c-reactive protein (CRP), interleukin- 6 (IL-6), lactate dehydrogenase (LDH), and ferritin. Those patients are old and have co-morbidities at baseline. Low-level serum albumin was reported in the patients who had worse complications [2–5].

Albumin is one of the major proteins found in the human body that serves as an anti-inflammatory agent [6, 7]. It binds with the reactive oxygen species (ROS) and reactive nitrogen species (RNS) produced during inflammation saving cellular injury [8]. Few studies found that low serum has a direct effect on mortality and worse complications in hospitalized COVID-19 patients [9–11].

So far, the studies have been mostly done on the patients who were hospitalized. Data on patients who presented to the emergency department (ED), urgent care (UC), or in the clinics and were discharged to self-quarantine is lacking.

We conducted this study to find out what percentage of patients needed hospitalization who were discharged from ED to self-quarantine, with the hypothesis that the patients with low serum albumin were at higher risk of hospitalization within three weeks of discharge from ED.

## Materials and methods

### Study design

A single-center, retrospective case-control study. The study was approved by the Cape Fear Valley Medical Center’s Institutional Review Board (IRB ID: 323–20).

### Study population

We included patients who presented to ED of a tertiary care hospital and had a discharge diagnosis of ‘COVID-19, virus identified’ (ICD 10 code U07.1) from March 01 to July 31, 2020. We reviewed 796 charts with severe acute respiratory syndrome coronavirus– 2 (SARS-CoV-2) detected on reverse transcriptase-polymerase chain reaction (RT-PCR) of the nasopharyngeal swab. A total of 342 patients were discharged from the ED. Out of those 342, the patients who were i) age ≥18 years old, and ii) had serum albumin documented were included in the study. The final study population included 112 patients.

The included patients were divided into normal serum albumin group (NSA) with serum albumin level of ≥3.5 g/dL and low serum albumin group (LSA) with serum albumin of <3.5 g/dL. They were followed up for a total of three weeks (21 days).

### Outcomes

The primary endpoint was the hospitalization due to any cause. The secondary outcome was hospitalization attributed to COVID-19 related symptoms (fever, cough, dyspnea, diarrhea, chest pain, and fatigue).

### Statistical analysis

A formal sample size calculation was not carried out as all patients meeting criteria in the pre-specified timeframe were included. The differences in categorical variables were analyzed using a Chi-square test, or Fisher’s exact test as appropriate. A Student t-test was utilized to evaluate differences in continuous variables that were normally distributed, and a Wilcoxon Rank Sum test was utilized if the data was not normally distributed. Continuous variables were expressed as mean ± SD and categorical variables were expressed as the frequency with percentages. The association of albumin level category and outcome was estimated in univariate and multivariate logistic regression analyses in terms of odds ratio (OR). A p-value of <0.05 was considered significant. Data analysis was performed using STATA 16.1 (Stata Corp, College Station, TX).

## Results

We included a total of 112 patients in our study. The LSA group had 65 patients and the NSA group had 47 patients. Baseline demographics and lab values based on albumin groups are outlined in Table 1. The LSA group had numerically higher frequency of female (60% vs 44%), and African American patients (52.31% vs 48.94%) as compared to the NSA group. The mean age of the LSA group was slightly higher (47.5 years old vs 42 years old) than the NSA group but was not statistically significant. The patients in the LSA had higher frequencies of co-morbidities as compared to the NSA group but were not statistically significant.

**Table 1:** Baseline characteristics of normal and low serum albumin groups.

	Normal Albumin, NSA (n = 47)	Low Albumin, LSA (n = 65)	p-value
Age, m (SD)	42 (15.54)	47.5 (17.80)	0.08
Female, n (%)	21 (44.68)	39 (60)	0.10
Race			0.88
White, n (%)	12 (25.53)	14 (21.54)	
African American, n (%)	23 (48.94)	34 (52.31)	
Others, n (%)	12 (25.53)	17 (26.15)	
Comorbid Conditions			
Diabetes Mellitus, n (%)	6 (12.77)	12 (18.46)	0.41
Hypertension, n (%)	23 (35.38)	11 (23.40)	0.17
CKD, n (%)	0 (0)	5 (7.69)	0.05
ESRD on HD, n (%)	0 (0)	0 (0)	n/a
COPD, n (%)	0 (0)	4 (6.15)	0.08
Other lung diseases, n (%)	4 (8.51)	7 (10.77)	0.69
CHF, n (%)	0 (0)	11 (1.54)	0.39
CAD, n (%)	2 (4.26)	5 (7.69)	0.45
HIV, n (%)	1 (2.13)	1 (1.54)	0.81
Malignancy, n (%)	2 (4.26)	3 (4.62)	0.92
Smoking, n (%)	9 (20.93)	7 (12.07)	0.22
Alcohol Dependency, n (%)	2 (4.26)	4 (6.15)	0.66
Obesity, n (%)	22 (46.81)	33 (50.77)	0.67
Cirrhosis, n (%)	0 (0)	0 (0)	n/a
Symptoms			
Onset of Symptoms, m (SD)	5.31 (5)	5.32 (4.35)	0.99
Fever, n (%)	21 (44.68)	34 (52.31)	0.42
Cough, n (%)	27 (57.45)	36 (55.38)	0.82
SOB, n (%)	19 (40.43)	36 (55.38)	0.11
Diarrhea, n (%)	8 (17.02)	15 (23.08)	0.43
Chest Pain, n (%)	12 (25.53)	7 (10.77)	0.04
Fatigue, n (%)	16 (34.04)	16 (24.62)	0.27
Laboratory Results			
Albumin (g/dL), m (SD)	3.60 (0.63)	3.12 (0.40)	<0.001
CRP (mg/L), m (SD)	15.6 (0.00)	40.92 (32.9)	0.02
Neutrophil (x 10^9^/L), m (SD)	4.09 (1.31)	6.30 (7.38)	0.17
Lymphocyte (x 10^9^/L), m (SD)	1.7 (0.94)	1.5 (0.98)	0.55
NL ratio, m (SD)	3.06 (1.92)	8.5 (21.36)	0.23
Platelets (x 10^9^/L), m (SD)	235.19 (62.17)	243 (93.92)	0.59
LDH (mg/dL), m (SD)	260 (0)	299 (113.42)	0.31

m = mean; n = number. Onset of symptoms in days. CKD = Chronic Kidney Disease; ESRD = End Stage Renal Disease; HD = Hemodialysis; COPD = Chronic Obstructive Pulmonary Disease; CHF = Congestive Heart Failure; CAD = Coronary Artery Disease; HIV = Human Immunodeficiency Virus; SOB = Shortness of Breath; CRP = C-Reactive Protein; NL = Neutrophil to Lymphocyte; LDH = Lactate Dehydrogenase.

### Primary outcome

Out of 112 patients, 12 (10.71%) were hospitalized within three weeks. The LSA group had 10 (15.38%) vs 2 (4.26%), p = 0.06 hospitalizations as compared to the NSA group (Table 2).

**Table 2:** Hospitalization in normal and low albumin groups (n = number).

Outcomes	Normal Albumin, NSA (n = 47)	Low Albumin, LSA (n = 65)	p-value
Hospitalization, n (%)	2 (4.26)	10 (15.38)	0.06
COVID-19 related hospitalization, n (%)	2 (4.26)	6 (9.23)	0.31

### Secondary outcome

In total 8 (7.14%) hospitalizations were attributed to COVID-19 related symptoms. The LSA group had 6 (9.23%) vs 2 (4.26%), p = 0.31 hospitalizations as compared to the NSA group that were attributed to COVID-19 related symptoms.

Logistic regression analysis did not reveal any difference in the odds of hospitalization in the NSA group during univariate (OR 0.24, 95% CI 0.05–1.17, p = 0.07) as well as multivariate analyses (OR 0.26, 95% CI 0.03–1.92, p = 0.19) (Table 3). The multivariate analysis revealed that the strong risk factors like diabetes mellitus (DM) (OR 3.2, 95% CI 0.55–19.02, p = 0.19) and obesity (OR 0.28, 95% CI 0.43–1.85, p = 0.18) did not influence the primary outcome (Table 3). A similar trend was seen in the analysis of secondary outcome (Table 4).

**Table 3:** Results from Multivariate Analysis (MV) for hospitalizations in the groups with normal and low albumin levels.

Hospitalization	Odds Ratio	95% CI	p-value
Albumin (ref Low Albumin)	0.26	0.03–1.92	0.19
Age	1.02	0.96–1.09	0.46
Sex	0.25	0.03–2.04	2.04
Race			
White	1.09	0.14–8.07	0.93
Others	01.38	0.11–17.33	0.80
Smoking	1		
DM	3.2	0.55–19.02	0.19
HTN	1.37	0.17–11.03	0.76
CKD	1	-	n/a
ESRD on HD	1	-	n/a
COPD	1	-	n/a
CHF	1	-	n/a
CAD	0.70	0.01–28.51	0.80
HIV	1	-	n/a
Malignancy	1	-	n/a
Alcohol Dependency	9.03	0.53–153.96	0.12
Obesity	0.28	0.43–1.85	0.18
Other Lung Diseases	1.58	0.11–21.48	0.73
Cirrhosis	1	-	n/a

MV controlled for Age; Sex; Race; Smoking; DM = Diabetes Mellitus; HTN = Hypertension, CKD = Chronic Kidney Disease; ESRD = End Stage Renal Disease; HD = Hemodialysis; COPD = Chronic Obstructive Pulmonary Disease; CHF = Congestive Heart Failure; CAD = Coronary Artery Disease; HIV = Human Immunodeficiency Virus; Malignancy; Alcohol Dependency; Obesity, Other lung Diseases; Cirrhosis.

**Table 4:** Results from Multivariate Analysis (MV) for COVID-19 related hospitalization in the groups with normal and low albumin levels.

COVID-19 related Hospitalization	Odds Ratio	95% CI	p-value
Albumin (ref Low Albumin)	0.62	0.07–5.11	0.66
Age	1.07	0.99–1.16	0.07
Sex	0.26	0.02–2.63	0.25
Race			
White	0.67	.06–7.19	0.74
Others	1.11	0.05–20.85	0.94
Smoking	1	-	n/a
DM	3.5	0.48–26.94	0.21
HTN	0.86	0.08–8.83	0.90
CKD	1	-	n/a
ESRD on HD	1	-	n/a
COPD	1	-	n/a
CHF	1	-	n/a
CAD	1	-	n/a
HIV	1	-	n/a
Malignancy	1	-	n/a
Alcohol Dependency	4.4	0.10–179.74	0.43
Obesity	0.60	0.07–5	0.63
Other Lung Diseases	2.6	0.15–43.70	0.5
Cirrhosis	1	-	n/a

MV controlled for Age; Sex; Race; Smoking; DM = Diabetes Mellitus; HTN = Hypertension, CKD = Chronic Kidney Disease; ESRD = End Stage Renal Disease; HD = Hemodialysis; COPD = Chronic Obstructive Pulmonary Disease; CHF = Congestive Heart Failure; CAD = Coronary Artery Disease; HIV = Human Immunodeficiency Virus; Malignancy; Alcohol Dependency; Obesity, Other lung Diseases; Cirrhosis.

## Discussion

In this single-center study, we found that more than 10% of the patients discharged from ED to self-quarantine needed hospitalization within three weeks. The patients who had normal serum albumin were less likely to need hospitalization after discharge, although the relationships were not statistically significant. The patient with normal serum albumin had fewer co-morbidities compared to patients with low serum albumin.

The LSA group had numerically higher frequencies of diabetes mellitus (DM), obesity, and congestive heart failure (CHF), but the data was clinically insignificant. We noticed that the LSA group had higher levels of inflammatory markers like CRP, LDH, and Neutrophil to Lymphocyte (NL) ratio which were also seen in previous studies where low serum albumin was associated with poor outcomes, though our findings were not statistically significant [12, 13]. Obesity, DM, and CHF are considered chronic inflammatory states [14, 15]. Touma et al. in their study concluded that low albumin had increased the risk of rehospitalization, and mortality. They also reported that patients with low albumin had higher number of co-morbidities at baseline [16]. The findings of our study are consistent with these prior findings. Akirov et al. reported that low serum is not only associated with higher mortality, but normalization of serum albumin decreased the rehospitalization and mortality. The state of chronic elevation of inflammatory markers in patients with such co-morbidities might have caused the lowering of serum albumin which is the main plasma anti-antioxidant, and hence signifying severity of COVID-19 [17].

The low albumin has been associated with adverse outcomes in various other disease conditions [18–23], as well as in COVID-19 patients who were hospitalized [9, 10, 24]. Kheir et al. concluded in their study that higher albumin level on admission was associated with favorable outcomes in hospitalized COVID-19 patients [11]. We hope this study will help understand the characteristics of COVID-19 who did not require hospitalization. Also, our study will help providers realize that patients with low albumin at presentation might be at higher risk of getting hospitalized in the future.

One of the limitations of our study is that it was a single-center study. The sample size was also small limiting the robustness of our statistical inference. Also, there was a lack of laboratory data for analysis, which could be due to the ED providers not ordering basic inflammatory markers in patients who did not clinically look sick to the extent of requiring hospitalization. Nevertheless, to the best of our knowledge, this is the first study that has explored the hospitalization of COVID-19 patients that were initially discharged to self-quarantine.

## Conclusion

In this single-center study, the low serum albumin was not associated with the risk of hospitalization in COVID-19 patients who were initially discharged to self-quarantine from the emergency department.

## Supporting information

S1 Dataset (ZIP)
