# Correction: Low serum albumin and the risk of hospitalization in COVID-19 infection: A retrospective case-control study

**DOI:** 10.1371/journal.pone.0296236

**Published:** 2023-12-18

**Authors:** Roshan Acharya, Dilli Poudel, Aakash Patel, Evan Schultz, Michael Bourgeois, Rishi Paswan, Scott Stockholm, Macylen Batten, Smita Kafle, Amanda Atkinson, Hafiz Sarwar

The eighth author’s name is spelled incorrectly. The correct name is: Macelyn Batten.
